# Hypoxia in Breast Cancer—Scientific Translation to Therapeutic and Diagnostic Clinical Applications

**DOI:** 10.3389/fonc.2021.652266

**Published:** 2021-03-11

**Authors:** Ying Zhang, Hongyi Zhang, Minghong Wang, Thomas Schmid, Zhaochen Xin, Lora Kozhuharova, Wai-Kin Yu, Yuan Huang, Fengfeng Cai, Ewelina Biskup

**Affiliations:** ^1^Department of Breast Surgery, Fudan University Shanghai Cancer Center, Shanghai, China; ^2^Department of Oncology, Shanghai Medical College, Fudan University, Shanghai, China; ^3^Department of Breast Surgery, Yangpu Hospital, Tongji University School of Medicine, Shanghai, China; ^4^Department of Health Management, Shanghai Public Health Clinical Center, Shanghai, China; ^5^Department of Medical Oncology, St. Claraspital, Basel, Switzerland; ^6^Department of General Surgery, Zhongshan Hospital, Fudan University, Shanghai, China; ^7^Faculty of Medicine, University of Freiburg, Freiburg, Germany; ^8^Cellomics International Limited, Hong Kong, China; ^9^Division of Internal Medicine, University Hospital of Basel, University of Basel, Basel, Switzerland; ^10^Department of Advanced Biomedical Sciences, Federico II University of Naples, Naples, Italy

**Keywords:** hypoxia, breast cancer, hypoxia-induced factors, biomarkers, oxidative stress, hypoxia-related treatment, precision medicine

## Abstract

Breast cancer has been the leading cause of female cancer deaths for decades. Intratumoral hypoxia, mainly caused by structural and functional abnormalities in microvasculature, is often associated with a more aggressive phenotype, increased risk of metastasis and resistance to anti-malignancy treatments. The response of cancer cells to hypoxia is ascribed to hypoxia-inducible factors (HIFs) that activate the transcription of a large battery of genes encoding proteins promoting primary tumor vascularization and growth, stromal cell recruitment, extracellular matrix remodeling, cell motility, local tissue invasion, metastasis, and maintenance of the cancer stem cell properties. In this review, we summarized the role of hypoxia specifically in breast cancer, discuss the prognostic and predictive value of hypoxia factors, potential links of hypoxia and endocrine resistance, cancer hypoxia measurements, further involved mechanisms, clinical application of hypoxia-related treatments and open questions.

## Introduction

Breast cancer has been the most commonly diagnosed cancer and the leading cause of cancer deaths among women, accounting for ~630,000 deaths in 2018 (1). It is a highly heterogeneous disease and clinic-pathological factors as well as multi-genomic assays allow for a sub-classification into several types and diverse subtypes, with different biological features and prognoses as well as different response to treatments (2, 3). Breast cancer mortality has decreased since the early 1990s (absolute reduction of 39% from 1989 to 2015) due to a combination of improved prevention, screening and earlier detection/diagnosis, lifestyle changes and awareness, as well as significant improvements in anti-cancer therapies. Despite these advances, worldwide every minute a woman dies from breast cancer. While mortality has been decreasing the incidence of breast cancer has been increasing. In the US, every 2 min and in the EU, 8 women are newly diagnosed every hour (4). China is another giant, modern society, where the burden of cancer is reaching epidemic proportions. In 2015, China National Cancer Center reported 12,000 per day (4.3 million) newly diagnosed cancer cases, accounting for a quarter of the global prevalence, out of which 15% are attributed to breast cancer (in women) (5).

Hypoxia is a characteristic feature of cancer. Tissue microenvironment influences tumorigenesis and tumor progression. Most solid tumor types have been shown to exhibit regions of hypoxia. The presence of a hypoxic microenvironment is a recognized event in mutagenesis and cancer development. At the same time, cancer *per se* also induces hypoxia secondary to inflammation (6, 7). It is crucial to discern the term hypoxia, which is mostly understood as a general system state which can accompany any advanced malignancy via completely different mechanisms (e.g., pathways reactive to necrosis, apoptosis, chronic permanent inflammation). The local hypoxia, however, is intentionally produced by tumor cells in order to induce angiogenesis and growth factors directed to tumor growth and metastatic features, with the healthy tissue surrounding the tumor experiencing an accompanying great structural and functional damage.

At the present time, various studies demonstrated a correlation between hypoxia and carcinogenesis, metastasis, treatment failure, and patient mortality (8–10). About 25–40% of invasive breast cancers exhibit hypoxic regions (11). Local hypoxia within the tumor and surrounding microenvironment is mainly a result of an abnormal anatomy of blood vessels, excessive angiogenesis leading to local obstructions or compressions and disturbed microcirculation (12). Generation of a hypoxic environment and the activation of its main effector, the hypoxia-inducible factor (HIF)-1, are even more common features of advanced malignancy (13, 14).

Hypoxia may retain cancer stem cells in their undifferentiated state, permitting solely cancer cells to differentiate and uninterruptedly accumulate genetic and epigenetic changes over a period of time (15, 16). Intra-tumoral hypoxia increases the number of breast cancer stem cells (BCSCs), which are essential for disease progression and recurrence (8, 17). Hypoxic breast cancer (and other) tumors are associated with a more aggressive phenotype, increased risk of metastasis and resistance to anti-cancer treatments (18, 19).

In this review, we address the role of the local hypoxia in breast cancer. We discuss unanswered questions and potential hypoxia-related treatments, in the context of relevant published literature.

## Hypoxia-Inducible Factors (HIFs) and Breast Cancer

The response of cancer cells to hypoxia is principally ascribed to HIFs, which are composed of a HIF-α (HIF-1α, HIF-2α, or HIF-3α) and a HIF-1ß subunit (8, 20, 21). These HIFs are responsible for the majority of the hypoxia-induced changes in gene expression (22, 23). HIF-1α-mediated mechanisms favor tumor growth and malignant progression, up- and down-regulation of genes, as well as pathologic modifications of the genome (24), whereas HIF-2α stimulates some, but not all genes activated by HIF-1α (25). HIF-1α responds in transient manner to severe hypoxia with rapid stabilization and activation of target genes, whereas HIF-2α responds to moderate levels of hypoxia and accumulates over time (26).

Under normoxic conditions, HIF1α is degraded by the proteasome, while under hypoxia, it translocates to the nucleus and forms a heterodimer with HIF-1β, which triggers the hypoxic response- a coordinated gene expression program (27). The hypoxic response triggers a decrease in cellular metabolism, thus inactivating the mammalian target of rapamycin (mTOR) pathway (28).

In addition, HIFs activate the transcription of a large battery of genes encoding proteins that promote primary tumor vascularization and growth, stromal cell recruitment, extracellular matrix remodeling, cell motility, local tissue invasion, metastasis, HIF-1α promotes primary breast cancer growth, vascularization (8, 29). Overexpression of HIF in breast cancer was often proposed as an unfavorable feature (30).

Hypoxia and the expression of hypoxia-mediated proteins, such as HIF-lα and VEGF, have been suggested to be negative prognostic and predictive factors, owing to its multiple contributions to chemo- and radioresistance, angiogenesis, invasiveness, metastasis, resistance to cell death, altered metabolism, and genomic instability (10). As reported in a variety of studies, HIF-1α overexpression is significantly correlated to adverse outcomes and a poorer survival in breast cancer patients (31–34). Increased concentrations of HIF-1α have also been independently associated with a worse outcome, as demonstrated by immunohistochemistry in subsets of biopsies analyzed from both lymph node-negative (32) and lymph node-positive breast cancer patients (33, 35). Moreover, higher levels of both HIF-1α or HIF-2α in breast cancer biopsies are associated with metastasis to regional lymph nodes and distant organs, primary mammary tumor growth, as well as with an increased patient mortality (36, 37). The proposed mechanisms involve tumor-infiltrating cells (TICs). HIF-1α expression rises alongside tumor grading, being higher in less differentiated than in well-differentiated lesions (38). Finally, HIF-1α was proposed as a prognostic marker for an unfavorable outcome in those with T1/T2 tumors and positive axillary lymph nodes (29).

HIF-1β has also been reported to correlate with more aggressive cancer characteristics and poor survival, but the difference was not statistically significant in multivariate analysis (39).

## Hypoxia in Breast Cancer Development and Progression

Hypoxia plays an important role in tumor progression and development (13). Related processes include the meditation of angiogenesis, apoptosis, the glycolytic shift and the recruitment of tumor-associated macrophages (40).

Angiogenic growth factors and their receptors are significantly up regulated in response to hypoxia, which causes vascular effects including endothelial cell migration with increased vascular permeability and promotes tumor angiogenesis (41). During these phases of transformations and growth, as the vessels are loosening their hierarchy and become arbitrarily arranged, cancer and stromal cells have a restricted access to nutrients and oxygen. Oxygen partial pressure in the tumor is significantly lower than in the healthy tissue at the tumor margins. The tumor cells in the vicinity of perfused vessels obviously still benefit from their oxygen supply, while cells at a greater distance are particularly hypoxic. This exacerbates even more in anemic cancer patients. Moreover, when a region of hypoxia is encountered, tumor-associated macrophages (TAMs) are induced to accumulate and exhibit a tumorigenic phenotype. TAMs can also secrete angiogenic growth factors and are associated with angiogenesis and poor prognosis in invasive breast cancer (42).

Coordinated regulation of a number of pro- and anti-apoptotic pathways by both HIF-dependent and HIF-independent mechanisms governs susceptibility to hypoxia-induced apoptosis in a cell-type-specific manner (43).

Hypoxia inhibited the pro-apoptosis effects of serum deprivation, reduced the bax/bcl-2 ratio, decreased cytochrome c release and caspase 3 activity via induction of vascular endothelial growth factor (VEGF) (44). Also, hypoxia selects for p53^mut^ cells with elevated levels of apoptosis inhibitor bcl-2. The reduced ratio of p53/bcl-2 acts increases mutation rates within a clone population, promoting the oncogenesis of breast cancer (45, 46). Normally, such hypoxic state, if persistent, causes apoptosis of healthy cells, while some tumor cells stop dividing, but continue to exist and others (with a certain genetic predisposition) succeed in surviving and continue to be destructive. Out of many tumor cell populations, mechanism of selection will lead to a preference of those capable to assimilate and thrive under hypoxic conditions. These are particularly aggressive as they usually become apoptosis-resistant and thus the responsiveness to radiation or chemotherapy is reduced.

In addition, hypoxia maximizes the efficiency of the glycolytic shift via changes in the expression of glycolytic enzymes (47) and glucose transporter genes (48). Both the maximal glucose uptake and high efficiency of glucose utilization lay the basis for glycolytic respiration, which enables tumor cells to grow and proliferate under such conditions.

Extracellular matrix (ECM) is a network of proteins and proteoglycans, which supports diverse cellular functions (49). Apart from the direct increase on endothelial cells (ECs) via the expression of matrix metalloproteinase (MMPs) (50), ECM is also involved during hypoxia-driven angiogenesis (51). Numerous studies highlighted that hypoxia regulates the expression and stability of ECM proteins (collagen I and IV and laminin) in cancer cells (52). ECM deposited from co-cultures of Neonatal Fibroblasts (NuFF) with breast cancer cells supported 3-dimensional vascular morphogenesis (53, 54). Hypoxic fibers occupied a greater percent area and possessed larger diameter fibers than those deposited by co-cultures in normoxic conditions (51). It has been reported that HIF-1α is related to the changes in fiber organization, given that fiber alignment was abrogated in hypoxia-treated fibroblasts when HIF-1αwas knocked down (55). Overall, a disturbed and overloading structure results. This activates angiogenic responses by promoting up-regulated expression of vascular pro-angiogenic factors VEGFAand Ang1, proteolytic enzymes MT1-MMP, and MMP1, while leading to a down-regulation of the vascular destabilizing factor, thus altering EC responses (51). In sum, not only the architecture, content and order of the EC are modified, but the functional aspects as well.

Hypoxia and vascularity of the tumor are interrelated on various levels, including a variety of environmental and signaling components described above (56). Under hypoxia, a number of cells under lactic acid fermentation metabolism (anoxic), numerous messenger substances, including VEGF, stimulate afferent blood vessels to grow from neighboring tissues to tumor cells. Since a tumor cannot grow larger than 1 mm without neovascularization, a large number of disturbed vessels are being mobilized, to assure that the supply of nutrients to cancer cells is not relying exclusively via diffusion and the supply of oxygen the center of the grown cell cluster is adequate. Overall, different areas of a tumor are supplied with different levels of oxygen: Cancer cell clusters in place and detached CTCs with a lack of oxygen have comparatively few blood vessels. At the same time, as compared to clusters with normal oxygen content, the hypoxic clusters are significantly more aggressive, metastasizing more quickly. Thus, it becomes obvious that in order to improve the oxygen supply, the formation of blood vessels is stimulated around the primary tumor, while local hypoxia is tumor-induced in order to promote CTC detachments and thus metastasis.

## Hypoxia Prognostic Factors in Breast Cancer

Several recent studies have shown independent prognostic significance of a number of hypoxia related factors, such as PGC1, transcription intermediary factor 1γ (Tif1γ) or transforming growth factor -β (TGF-β), similarly to HIF1α, where this has been confirmed before (39).

TGFβ has been shown to have both tumor suppressive (early stages) and oncogenic (later stages, pro-metastatic and pro-EMT) effects. Especially the isoform TGFβ1 is an inhibitor of mammary gland epithelial cell proliferation and plays an important role in breast carcinogenesis.

TIF1γ contributes to breast cancer by controlling TGF-β/Smad signaling, leading to a TGF-β-induced EMT. A link was reported between TIF1γ and HIF1α in TNBC. In a study in press, we were able to show that the levels of Tif1γ were significantly lower in patients with breast cancer than in healthy controls. The average concentration of Tif1γ-discriminated between Tif1γ-positive and Tif1γ-negative patients. The latter group had a significantly worse OS (*P* = 0.0174); this was confirmed in the multivariate analysis. Tif1γ plasma level seems to be thus an independent prognostic factor for patients with breast cancer. This supports the potential of using measurements of Tif1γ plasma level to guide breast cancer therapy and monitoring.

Other proteins involved in cell homeostasis might become additional biomarkers in the early detection/diagnosis and monitoring of breast cancer if their apoptotic features react to the influence of aerobic vs. anaerobic microenvironment. Further studies are required to identify and validate new easily detectable, non-invasive biomarkers with prognostic power—studies of some such biomarkers are already ongoing, e.g., PGC1α, Tif1γ, etc.).

Increased levels of PGC1α were, similarly to HIF-1α, associated with more aggressive tumors -e.g., histologically higher grade and higher stage–and were therefore proposed as a prognostic marker for unfavorable outcomes, especially in positive axillary lymph nodes tumors (39). Recently, PGC1α was confirmed to be an independent prognostic marker, where over-expression correlates with poorer outcome in an unselected (all stages) breast cancer population (39).

All these markers (PGC1α, HIF-1α, and Tif1γ) can be measured easily from patients' plasma, which allows a simple, cost- and time-effective method for an improved clinical decision-making regarding treatment or even an early diagnosis for patients.

## Hypoxia and Breast Cancer Metastases

Despite the rapid progress in breast cancer treatment, the development of metastases remains the primary reason for breast cancer mortality (57). It is a complex process, which until now is known as a series of steps: epithelial-mesenchymal transition (EMT), local tissue invasion and intravasation, extravasation and metastatic niche formation (58). As mentioned above, hypoxia contributes to cell transformations, so that they undergo fundamental functional and structural changes. In this manner, a number of those cells, who were previously sedentary cancer cells, acquire properties that are essential for mobilization, conspicuously due to genetic modifications in p53 (tumor suppressor) and modifications on chromosome level (e.g., in Chromosome 1).

EMT is characterized by cellular and molecular changes that include loss of cell-to-cell adhesions (58). Thus, phenotypically, the migratory cells possess little or no adhesion molecules, while they develop a battery of lytic enzymes to invade lymphatic and blood vessels. HIF-1 activates EMT through regulating associated signaling pathways, modulating EMT-associated inflammatory cytokines, as well as interfering in other pathways, such as epigenetics (here, concrete data are still missing) (59). Transcription factors like E-cadherin, SNAIL (zinc finger protein snail), ZEB1 (zinc finger E-box-binding homeobox 1), and TWIST are also involved in the HIF-1 induced EMT (60) (Table 1).

**Table 1:** Role of various factors in breast cancer and hypoxia.

**Factor**	**Role in breast cancer**	**Role in hypoxia**
HIF-1α/ HIF-2α (Hypoxia-inducible factors) P4HA1, P4HA2	1. HIF-1α favor tumor growth and malignant progression, up- and down-regulation of genes, as well as pathologic modifications of the genome (24), whereas HIF-2α stimulates some, but not all genes activated by HIF-1α (25). 2. HIFs activate the transcription of a large battery of genes encoding proteins that promote primary tumor vascularization and growth, stromal cell recruitment, extracellular matrix remodeling, cell motility, local tissue invasion, metastasis, and maintenance of the cancer stem cell properties (29, 61) 3. Up-regulating the expression levels of pro-collagen prolyl (P4HA1 and P4HA2) and lysyl hydroxylases (PLOD2) 4. HIF-1α promotes primary breast cancer growth, vascularization and metastases to axillary lymph nodes and distant organs (61)	1. HIFs are responsible for the majority of the hypoxia-induced changes in gene expression (23, 62) 2. HIF-1α responds in transient manner to severe hypoxia with rapid stabilization and activation of target genes, whereas HIF-2α responds to moderate levels of hypoxia and accumulates over time (26)
MMPs (matrix metalloproteinase)	MMPs degrade many of the components of the ECM, which enables cancer cells to invade the surrounding tissues and intravasation	Hypoxia and HIF-1 up-regulate the expression levels and/or MMP-2 and MMP-9 (63, 64)
LOX, LOXL2, LOXL4 (lysyl oxidase)	ECM remodeling in the primary and the distant sites -> metastatic niche formation (65)	Produced by hypoxic breast cancer cells
Protein kinase C-δ, transcription factor STAT3, interleukin IL6, and NANOG	Essential for BCSC	As a linkage between obesity and breast cancer via hypoxia
PGC1 (peroxisome proliferator-activated receptorγcoactivator-1)	Prognostic and predictive marker, associated with more aggressive tumor characteristics and poorer outcomes (39)	An hypoxia factor with independent prognostic significance
Tif1γ (transcription intermediary factor 1γ)	TIF1γ contributes to breast cancer by controlling TGF-β/Smad signaling, leading to a TGF-β-induced EMT	An hypoxia factor with independent prognostic significance
TGF-β (transforming growth factor -β)	Tumor suppressive (early stages) and oncogenic (later stages, pro-metastatic and pro-EMT) effects	An hypoxia factor with independent prognostic significance
E-cadherin, SNAIL (zinc finger protein snail), ZEB1 (zinc finger E-box-binding homeobox 1), and TWIST	These factors are involved in the HIF-1 induced EMT (60)	Having some relationship with hypoxia induced EMT

MMPs degrade many of the components of the ECM, which enables cancer cells to invade the surrounding tissues and intravasation. Hypoxia and HIF-1 up-regulate the expression levels and/or MMP-2 and MMP-9 (63, 64), which are positively correlated with a higher incidence of metastases and with a poor prognosis (64). HIF-1αplays a critical role in collagenogenesis by up-regulating the expression levels of pro-collagen prolyl (P4HA1 and P4HA2) and lysyl hydroxylases (PLOD2), which are reported to be crucial for breast cancer metastasis (55).

Hypoxic breast cancer cells produce multiple members of the lysyl oxidase (LOX) family, including LOX, LOXL2, and LOXL4, in a HIF-1-dependent manner (65). LOX remodel ECM both in the primary and the distant site, which provokes metastatic niche formation (66). Furthermore, HIF-1 induces miR-210-expression, a non-coding RNA, which contributes to tumor proliferation and forming of metastasis, alongside of other non-coding RNAs (miRNAs and lncRNAs) (67).

## Hypoxia and Breast Cancer Stem Cells

Breast Cancer Stem Cells (BCSCs) are characterized by an unlimited self-renewal differentiation potential, performance of symmetrical and asymmetric cell divisions, as well as regeneration (68). In mesenchymal stem cells (MSC), it was shown that most of them are exposed to a lower oxygen concentration *in vivo*, e.g., by about 7% in the medulla or adipose tissue. *Ex vivo*, culturing of MSC under hypoxic oxygen concentrations resulted in a higher growth rate, glucose consumption and longer life at a constant level of the stem cell functionality (69–71).

The metastasis-promoting effects of HIF-1 help to maintain an expanding renewing population of BCSCs ready to be distributed much like seeds or pollen blowing in the wind (72). Increased expression of HIF-1α and HIF-2α in BCSCs lead to increased expression of pluripotency factors such as NANOG, OCT4, SOX2, and KLF4 in response to intratumoral hypoxia (73). HIFs also mediate complex and bidirectional paracrine signaling between breast cancer cells (BCC) and MSC that stimulate breast metastasis. Interactions between BCC and MSC are supposedly mediated by CXCL10→CXCR3, CCL5→CCR5, and PGF→VEGFR1 signaling in a HIF-dependent manner (66). Further research is needed to explore these interdependencies more fully.

## A Link Between Obesity and Breast Cancer Via Hypoxia?

Despite numerous epidemiological studies illustrating the link of obesity and breast cancer, the underlying mechanisms are not elaborated. In addition, no general statements about the cancer-and-obesity relation can be made, as seen in the complexity of the breast cancer: e.g., postmenopausal obesity is correlated with an increased risk of breast cancer. Such a clear association has not been proven for obese in premenopausal females (74). Longitudinal data regarding the relapse rate link to obesity are still suboptimal, but from epidemiological observations, conclusions were already drawn and sufficient evidence is now available that a healthy body weight has a positive effect on the survival of breast cancer patients (75).

A potential causal relation is given based on the local hypoxia in breast cancer via the adenosine receptor 2B (A2BR), a modulator of glucose homeostasis and obesity (76). Elevated A2BR expression have been found in adipose tissue of obese individuals. Under hypoxic conditions in the breast cancer tissue (A2BR) is overexpressed. A2BR is linked both to adipositas and to BCSC. It primarily regulates pre-adipocyte differentiation and macrophage inflammation in adipose tissue. When activated (among others through HIF1 factors), A2BR leads to the activation of protein kinase C-δ, transcription factor STAT3, interleukines IL6, and NANOG. The 2 latter mediators are essential for production of BCSC, thus tumorigenesis and growth, as well as recurrence. Experiments *in vitro* showed that both a drug-related or genetic inhibition of A2BR expression or functionality lead to a decrease in BCSC enrichment, significantly reducing the tumor initiation and metastasis (68). These findings are fundamental to understand the known link between obesity and more aggressive breast cancer characteristics, as well as the higher risk of developing postmenopausal breast cancer.

## Hypoxia and Treatment Resistance

Hypoxia is known to directly or indirectly confer resistance to irradiation, some chemotherapeutic drugs, and endocrine therapy (24). Hypoxic tumors are less responsive to radiation therapy, mainly because the lack of oxygen causes DNA damage (77). Moreover, the responsiveness of malignancy to chemotherapeutic agents is modulated by reducing the susceptibility to DNA damage, inducing cell cycle arrest and limiting drug delivery under poor perfusion (10). The activation of ROS-shielding pathways (78) and overexpression of anti-apoptosis genes (79) mediated by hypoxia contribute to taxane resistance. As for ER-positive breast cancer patients, hypoxia has been shown to down-regulate ERα in several breast cancer cell lines and to influence the responsiveness to tamoxifen (38, 80). SNAT2, an amino acid transporter, was regulated by both ERα and HIF-1α (predominantly), leading to endocrine resistance under hypoxia (81).

While suppressing VEGF pathway initially decreases tumor progression rate and vasculature density, the activation of interrelated pathways and signaling molecules following VEGF blockade compensates the insufficiency of VEGF and the initially blocked angiogenesis, explaining part of the failure observed with bevacizumab monotherapy (82).

## Future Measuring Hypoxia in the Breast

An innovative method was tested in Austria: the possibility of hypoxia measurement in the breast using magnetic resonance imaging (MRI). This opens up new avenues of research into hypoxia although at this stage MRI has clearly not been established in the clinical approach. Besides measuring oxygen content, the MRI can also assess neovascularization in breast tumors. A significant benefit of this approach would be lower costs and greater availability compared to PET or near-infrared spectroscopy. Advanced quantitative blood oxygenation level dependent (qBOLD) imaging can directly quantify the tissue oxygen tension, while vascular architectural mapping (VAM) measures the microvascular vessel diameter and architecture (83). This approach looks potentially promising but further research is clearly required to validate any clinical utility.

## Gene Expression and Hypoxia in Breast Cancer

The adaption to hypoxia is governed by multiple transcriptional and post-transcriptional changes in gene expression. Up to 1.5% of the human genome is estimated to be transcriptionally responsive to hypoxia (84). Recent years brought insights into various additional genes and pathways that have been identified as being responsive to hypoxia and which might serve as prognostic or predictive markers, and even as novel therapeutic targets. Clustering genes are chosen for their expression pattern (85–87). Since increased activity of the HIF-1a pathway is related to a more profound intratumoral hypoxia in basal-like breast tumors compared with other subtypes, gene signatures might guide treatment decisions for potential application of anti-hypoxic drugs in the future (88).

Gene signatures reflect hypoxic response at a transcriptional level, whereas microRNAs regulate it at a post-transcriptional level. Comparative analysis of hypoxia-regulated miRNAs by gene expression profiles might add additional information to target-prediction algorithms (89). Despite rapid development, this area still needs further clinical validations.

## Anti-hypoxic Treatment

HIF-1α promotes primary breast cancer growth, vascularization and metastases to axillary lymph nodes and distant organs (8). Increased HIF-1 expression shows strong correlations with poor prognostic outcomes and low survival rates of breast cancer patients (90). Therefore, targeting the HIF pathway might provide an attractive strategy to treat hypoxic tumors. Agents that inhibit HIF-1α protein accumulation and demonstrate anti-tumor effects include the topoisomerase I inhibitor, topotecan, as well as the cardiac glycoside digoxin (65, 91–93).

Since HIF-1α can be induced by hypoxia-independent signaling pathways, such as motor, ERBB2 (HER2) and MAP kinase, the therapeutic benefits of targeting these pathways may also be partially explained by a decrease in HIF-1α levels (71). Especially triple-negative breast cancers (TNBCs) have a high HIF transcriptional activity and respond poorly to currently available therapies (94). Therefore, HIF inhibitors may be particularly useful in the treatment of TNBCs. Pre-clinical studies suggest that the combination of cytotoxic chemotherapy with drugs that inhibit hypoxia-inducible factors are very promising in this group of patients (8). HIF-1 inhibitors, such as digoxin and acriflavine, showed convincing potential therapeutic effects by decreasing primary tumor growth, vascularization, invasion and metastasis in breast cancer animal models (65, 92, 95). Adding digoxin to paclitaxel or gemcitabine leads to tumor regression in TNBC by blocking HIF-dependent transcriptional responses that promote the resistance of CSCs to chemotherapy (96).

Another aspect might be hypoxia-based treatments, where a synergetic effect with drugs that cause treatment-induced hypoxia, e.g., bevacizumab, is applied (8). Despite compelling evidence-linking hypoxia with treatment resistance and adverse prognosis, the activity of hypoxia-activated drugs also depends on the coincidence of tumor hypoxia, expression of specific prodrug-activating reductases and intrinsic sensitivity of malignant clones to the cytotoxic effector (97). Hypoxia-based drugs have been tested in clinical trials to further validate their efficacy in cancer treatment. However, the failure of 2 major clinical trial efforts (tirapazamine and evofosfamide) calls for further research (97). Hypoxia itself is highly variable between and within individual tumors and is not consistent among all breast cancer subtypes.

In the era of personalized precision medicine, clinical trials are warranted to determine whether anti-hypoxia drugs may increase the survival of breast cancer patients alone or in combination with current therapeutic regimens. It is pivotal to explore the decisive influence of hypoxia on the course of breast cancer in order to gain a deeper understanding of individual disease trajectories and to better forecast them. This knowledge can then be used in the future to develop and implement adequate therapy for each individual patient.

## Author Contributions

YZ, HZ, MW, TS, ZX, LK, W-KY, YH, FC, and EB contributed to the writing and editing of the manuscript. All authors contributed to the article and approved the submitted version.

## Conflict of Interest

W-KY and YH were employed by the company Cellomics International Limited. The remaining authors declare that the research was conducted in the absence of any commercial or financial relationships that could be construed as a potential conflict of interest.
